# Decompressive Craniectomy vs. Craniotomy Only for Traumatic Brain Injury: A Propensity-Matched Study of Long-Term Outcomes in Neuropsychology

**DOI:** 10.3389/fneur.2022.813140

**Published:** 2022-03-08

**Authors:** Zhengqian Guo, Wantao Ding, Dan Cao, Yong Chen, Jian Chen

**Affiliations:** ^1^Department of Neurosurgery, Tongji Hospital, Tongji Medical College, Huazhong University of Science and Technology, Wuhan, China; ^2^Wenzhou Seventh People's Hospital, Wenzhou, China

**Keywords:** decompressive craniectomy, craniotomy, traumatic brain injury, neuropsychology, long-term outcomes

## Abstract

**Background:**

Both decompressive craniectomy (DC) and craniotomy only (CO) are commonly performed to treat patients with traumatic brain injury (TBI) by evacuation of intracranial hemorrhage (ICH) and control intracranial pressure (ICP). The outcomes of these two procedures have been well-studied; however, most research studies have focused on physical functions. The purpose of our study is to assess long-term outcomes in neuropsychology after DC or CO in TBI patients.

**Methods:**

Information was collected from patients with TBI who had undergone DC or CO and were then in the postoperative stable phase (6–24 months after injury). Propensity scoring matched the patients in a 1:1 ratio for demographics, cause of injury, TBI subtype, TBI severity, computed tomography (CT) findings, surgery side, and interval from TBI. We used Wechsler Adult Intelligence Scale-Chinese Revision (WAIS-RC), Wechsler Memory Scale-Chinese Revision (WMS-RC), Physical Self-maintenance Scale (PSMS), Instrumental Activities of Daily Living Scale (IADL), and Glasgow Outcome Scale-Extended (GOSE) to measure the long-term outcomes in TBI patients, especially in neuropsychology.

**Results:**

There were 120 TBI patients included in our study. After matching, 74 patients were paired into the DC group (*n* = 37) and the CO group (*n* = 37). There were no differences in the gender (*P* = 1.000), age at injury (*P* = 0.268), marital status (*P* = 0.744), pre-injury employment (*P* = 0.711), comorbidities (*P* = 1.000), education level (*P* = 0.969), cause of injury (*P* = 0.357), TBI subtype (*P* = 0.305), Glasgow Coma Scale (GCS) total score (*P* = 0.193), unconsciousness (*P* = 0.485), traumatic subarachnoid hemorrhage (tSAH) (*P* = 0.102), unresponsive pupil (*P* = 1.000), midline shift (*P* = 0.409), cisterns compressed or absent (*P* = 0.485), surgery side (*P* = 0.795), and interval from TBI (*P* = 0.840) between the two groups. The CO group was associated with better cognitive function in WAIS-RC OIQ (*P* = 0.030) and WAIS-RC FIQ (*P* = 0.021) and better daily function in IADL (*P* = 0.028) and ADL total (*P* = 0.030). The DC group also had a lower GOSE (*P* = 0.004) score compared to the CO group. No difference was observed in WAIS-VIQ (*P* = 0.062), WMS-RC MQ (*P* = 0.162), and PSMS (*P* = 0.319).

**Conclusion:**

In the matched cohort, patients who underwent CO had better long-term outcomes in cognitive and daily function compared with DC. Future randomized control trials are needed for intensive studies on physical and neuropsychological prognosis in TBI patients.

## Introduction

Traumatic brain injury (TBI) is one of the major traumatic diseases that threaten human health. Worldwide, over 50 million people have a TBI each year (1). Besides paralysis, coma, and aphasia, the sequelae of various mental disabilities, such as cognitive impairment and personality changes caused by TBI, also bring a tremendous burden to the patients and their families.

Some patients with TBI require aggressive medical treatment to remove the intracranial hematoma to resolve the elevated intracranial pressure (ICP) because of the mass effect. There are two surgical strategies in which TBI is performed. Decompressive craniectomy (DC) is a procedure where part of the skull is removed to control the ICP, while cranial reconstruction is needed in the next few months with autologous bones or a synthetic implant. In craniotomy only (CO), the bone flap is returned after evacuation of the intracranial hematoma (ICH). Currently, both procedures are generally used by surgeons to treat TBI; the safety and efficacy of DC vs. CO is still controversial, and definitive guidelines are lacking. The neurosurgeon's experiences, which are based on the judgment of preoperative computed tomography (CT) and the degree of intraoperative brain injury and swelling, usually determine whether to perform DC or CO for each patient (2).

Several studies used the Glasgow Outcome Scale-Extended (GOSE) or Glasgow Outcome Scale (GOS) to measure the long-term outcomes between the DC and CO groups (3–6), which lack specificity and sensitivity, especially in patients' neuropsychology. For TBI patients, rehabilitation of mental health is just as essential as physical function in order to return to their pre-injury work and life. Apart from this, TBI might be a major risk factor for late neurodegenerative disorders such as dementia and Parkinson's disease (7). Thus, the purpose of our research was to compare the long-term neuropsychological prognosis of TBI patients treated with DC vs. CO. We hypothesized that TBI patients who had received CO would have better outcomes, especially in cognitive and daily function, than those who had received DC because of relatively less invasion and fewer postoperative complications.

## Patients and Methods

### Patient Population

This study included 120 TBI patients who underwent DC or CO from our center from 2019 to 2020. All patients were now in the postoperative stable phase (6–24 months after injury). The inclusion criteria were (1) age at injury ≥18 years, (2) patients can complete the neuropsychological assessment alone or with guidance, and (3) written informed consent provided by the patients or guardian. The exclusion criteria were (1) patients who underwent secondary DC, (2) patients who underwent surgery for posterior fossa, (3) DC patients without cranioplasty, and (4) patients in a vegetative state.

### Data Points

According to the previous study, there are multiple factors that contribute to the outcomes of TBI (8). Therefore, we retrospectively collected the following data points from medical records for each patient in order to create these two well-matched cohorts. These variables were patient demographics (including gender, age at injury, marital status, pre-injury employment, comorbidities, education level), cause of injury, TBI subtype, Glasgow Coma Scale (GCS) total score at pre-operation, unconsciousness, unresponsive pupil, accompanied by traumatic subarachnoid hemorrhage (tSAH), midline shift, cisterns compressed or absent, surgery side, and interval from TBI.

### Outcome Measures

All outcome measures were performed in the postoperative stable phase (6–24 months after injury).

#### Cognitive Functioning

Cognitive abilities were assessed with the Wechsler Adult Intelligence Scale-Chinese Revision (WAIS-RC) and the Wechsler Memory Scale-Chinese Revision (WMS-RC). The WAIS-RC contains a verbal intelligence quotient (VIQ), operation intelligence quotient (OIQ), and full intelligence quotient (FIQ), which can assess different cognitive domains. Meanwhile, the WMS-RC test was used to assess memory function with the memory quotient (MQ).

#### Daily Functioning

The Activity of Daily Living Scale (ADL) was divided into the Physical Self-maintenance Scale (PSMS) and the Instrumental Activities of Daily Living Scale (IADL) to assess patients' daily functioning. The former is the basis for maintaining somatic activities, while the latter includes a range of activities related to community activities, such as calling, shopping, traveling, and other activities that involve managing social interactions or environment variability (9). Higher scores indicate more severe functional impairment.

The GOSE has also been introduced in this study.

### Data Analysis

All statistical analyses were performed with the R software package (v4.0.3; R Foundation, Vienna, Austria). Continuous parametric data were reported as a mean ± standard deviation (M ± SD) and as a median with an interquartile range (M, IQR) for nonparametric data, whereas categorical variables were presented as numbers and percentages. We used the Mann–Whitney *U*-test and Student's *t*-test to explore differences between the two groups (DC and CO) for non-parametric and parametric continuous variables, respectively. We used the Pearson Chi-squared test or Fisher test to identify differences in two groups for categorical variables. The statistical significance was set as α = 0.05.

To minimize selection confounding bias, we used propensity score matching in a 1:1 ratio in the DC and CO groups. Factors such as patient demographics, cause of injury, TBI subtype, GCS total score at pre-operation, unconsciousness, unresponsive pupil, accompanied by tSAH, midline shift, cisterns compressed or absent, surgery side, and interval from TBI were used to conduct the match. The propensity score results in balancing all the covariates that are used to generate the score.

## Results

Finally, 120 TBI patients were enrolled in this study, with 70 who underwent DC and 50 who underwent CO. Of these 120 patients, 74 (DC, 37; CO, 37) were propensity score-matched in a 1:1 ratio.

The characteristics of the study population are demonstrated in Table 1. Before matching, variables related to the severity of injury and the CT findings, such as TBI subtype (*P* < 0.001), GCS total score (*P* < 0.001), unconsciousness (*P* < 0.001), accompanied by tSAH (*P* = 0.001), unresponsive pupil (*P* < 0.001), midline shift (*P* < 0.001), and cisterns compressed or absent (*P* = 0.002) were significantly different between the two groups. It follows that patients receiving DC had more severe head injury than their counterparts in the CO group. After matching, there were no differences in gender (*P* = 1.000), age at injury (*P* = 0.268), marital status (*P* = 0.744), pre-injury employment (*P* = 0.711), comorbidities (*P* = 1.000), education level (*P* = 0.969), cause of injury (*P* = 0.357), TBI subtype (*P* = 0.305), GCS total score (*P* = 0.193), unconsciousness (*P* = 0.485), accompanied by tSAH (*P* = 0.102), unresponsive pupil (*P* = 1.000), midline shift (*P* = 0.409), cisterns compressed or absent (*P* = 0.485), surgery side (*P* = 0.795), and interval from TBI (*P* = 0.840) between patients in the DC and CO groups. All the characteristics, including the severity of injury and the CT findings, were balanced by the match.

**Table 1:** Baseline characteristics for all TBI patients.

**Characteristic**	**Before matching**			**After matching**		
	**CO (*n* = 50)**	**DC (*n* = 70)**	***P*-value**	**CO (*n* = 37)**	**DC (*n* = 37)**	***P*-value**
Male sex	34 (68.0%)	44 (62.9%)	0.560	23 (62.2%)	23 (62.2%)	1.000
Age at injury, years	46 (36–55)	51 (40–58)	0.182	44 (37–55)	52 (37–61)	0.268
Married	41 (82.0%)	62 (88.6%)	0.309	31 (83.8%)	32 (86.5%)	0.744
Pre-injury employment	47 (94.0%)	63 (90.0%)	0.519	34 (91.9%)	32 (86.5%)	0.711
Comorbidities	8 (16.0%)	13 (18.6%)	0.715	5 (13.5%)	5 (13.5%)	1.000
Education level			0.431			0.969
Primary or less	25 (50.0%)	32 (45.7%)		17 (45.9%)	18 (48.6%)	
Middle	20 (40.0%)	25 (35.7%)		15 (40.5%)	14 (37.8%)	
High school or above	5 (10.0%)	13 (18.6%)		5 (13.5%)	5 (13.5%)	
Cause of injury			0.562			0.357
Fall	5 (10.0%)	5 (7.1%)		3 (8.1%)	3 (8.1%)	
Traffic	43 (86.0%)	64 (91.4%)		32 (86.5%)	34 (91.9%)	
Violence	2 (4.0%)	1 (1.4%)		2 (5.4%)	0 (0.0%)	
TBI subtype			<0.001^*^			0.305
ASDH	8 (16.0%)	30 (42.9%)		7 (18.9%)	9 (24.3%)	
Contusion	23 (46.0%)	35 (50.0%)		21 (56.8%)	24 (64.9%)	
EDH	19 (38.0%)	5 (7.1%)		9 (24.3%)	4 (10.8%)	
GCS total score	11 (9–13)	8 (5–10)	<0.001^*^	10 (8–13)	9 (8–13)	0.193
Unconsciousness	18 (36.0%)	51 (72.9%)	<0.001^*^	16 (43.2%)	19 (51.4%)	0.485
Accompanied by tSAH	37 (74.0%)	67 (95.7%)	0.001^*^	29 (78.4%)	34 (91.9%)	0.102
Unresponsive pupil						
One or none reactive	2 (4.0%)	25 (35.7%)	<0.001^*^	2 (5.4%)	3 (8.1%)	1.000
Midline shift, mm	7 (4–11)	11 (7–15)	<0.001^*^	8 (6–12)	8 (6–12)	0.409
Cisterns compressed or absent	19 (38.0%)	47 (67.1%)	0.002^*^	16 (43.2%)	19 (51.4%)	0.485
Surgery side			0.168			0.795
Left	23 (46.0%)	25 (35.7%)		15 (40.5%)	15 (40.5%)	
Right	22 (44.0%)	29 (41.4%)		17 (45.9%)	15 (40.5%)	
Bilateral	5 (10.0%)	16 (22.9%)		5 (13.5%)	7 (18.9%)	
Interval from TBI, months	11 (7–14)	10.0 (8–16)	0.532	11 (7–15)	9 (7–14)	0.840
Shunt placement	0	9 (12.9%)	0.010^*^	0	3 (8.1%)	0.240

* *Statistically significant*.

Table 2 shows the results of long-term outcomes in neuropsychology for the study populations before matching and after matching. Before matching, 120 patients (DC, 70; CO, 50) were enrolled in the analysis. There were significant differences between the DC and CO groups for each test. Specifically, the WAIS-RC VIQ (*P* = 0.006), WAIS-RC OIQ (*P* = 0.001), and WAIS-RC FIQ (*P* = 0.001) tests showed that patients who underwent DC had worse cognitive function. Meanwhile, according to the results of WMS-RC MQ (*P* = 0.019), patients in the DC group had more severe memory impairment compared to the patients in the CO group. The daily functioning was measured by the ADL scale and demonstrated higher scores in the DC group for the PSMS (*P* = 0.002), IADL (*P* = 0.001), and ADL total (*P* <0.001). At the last, patients in the CO group also had a higher GOSE (*P* <0.001) score than patients in the DC group. After matching, the results changed slightly. In terms of cognitive function, WAIS-RC OIQ (*P* = 0.030) and WAIS-RC FIQ (*P* = 0.021) remained significantly different between the DC and CO groups, with the patients who underwent CO having less cognitive impairment. However, they did not show significant statistical differences in the WAIS-VIQ (*P* = 0.062) test. Similarly, the results of WMS-RC MQ (*P* = 0.162), which represents memory function, were similar in both groups. In terms of daily functioning, patients who underwent CO had better daily living ability with a lower score in IADL (*P* = 0.028) and ADL total (*P* = 0.030). However, the PSMS (*P* = 0.319) did not show differences between these two groups. In addition, the DC group still had a lower GOSE (*P* = 0.004) score compared to the CO group at the time of measurement.

**Table 2:** Long-term neuropsychological outcomes for all TBI patients.

**Variable**	**Before matching**			**After matching**		
	**CO (*n* = 50)**	**DC (*n* = 70)**	***P-*value**	**CO (*n* = 37)**	**DC (*n* = 37)**	***P-*value**
WAIS-RC VIQ	76.16 ± 14.44	69.30 ± 12.30	0.006^*^	75.95 ± 15.00	69.78 ± 12.86	0.062
WAIS-RC OIQ	77.88 ± 12.51	70.47 ± 11.51	0.001^*^	77.00 ± 13.26	70.46 ± 12.07	0.030^*^
WAIS-RC FIQ	74.82 ± 12.94	67.20 ± 11.55	0.001^*^	74.46 ± 13.75	67.43 ± 11.86	0.021^*^
WMS-RC MQ	63.98 ± 12.80	58.43 ± 12.45	0.019^*^	62.51 ± 13.32	58.30 ± 12.31	0.162
ADL (PSMS)	6.0 (6.0–6.3)	7.0 (6.0–9.0)	0.002^*^	6.0 (6.0–7.0)	6.0 (6.0–8.0)	0.319
ADL (IADL)	12.0 (10.0–14.3)	15.0 (12.0–19.3)	0.001^*^	12.0 (10.0–14.5)	14.0 (11.5–18.0)	0.028^*^
ADL total	18.0 (16.0–20.5)	22.0 (18.8–29.0)	<0.001^*^	18.0 (16.0–21.0)	21.0 (17.5–26.5)	0.030^*^
GOSE	7.0 (6.0–7.0)	6.0 (5.0–6.0)	<0.001^*^	7.0 (6.0–7.0)	6.0 (5.0–6.5)	0.004^*^

* *Statistically significant*.

Before and after matching, there were nine (*P* = 0.010) and three (*P* = 0.240) patients who underwent DC with hydrocephalus requiring shunt placement. In contrast, none of the patients in the CO group required this procedure. However, no statistical difference was observed between the two groups in the cohort after matching.

## Discussion

In this study, we sought to find the differences in the long-term outcomes of TBI patients undergoing DC compared with CO in neuropsychology. The global outcomes of post-TBI have been well-studied, and it is known to correlate with age at injury, education level, pre-injury employment status, comorbidities, and injury severity (8). Therefore, we propensity-scored and matched all the possible patient characteristics that may have confounded the results, including demographics (including gender, age at injury, marital status, pre-injury employment, comorbidities, education level), cause of injury, TBI subtype, GCS total score, unconsciousness, unresponsive pupil, accompanied by tSAH, midline shift, cisterns compressed or absent, surgery side, and interval from TBI. Our study demonstrated that after balancing these covariates, the CO group had better neuropsychological outcomes than the DC group in cognitive and daily functioning. This is especially true for operational and community activities. In addition, patients who underwent CO had a higher score in GOSE, which is similar to previous studies (3, 4). However, there was no difference between the two groups in terms of impairment in memory and physical daily function.

Currently, strong evidence to support treatment guidelines and recommendations is scarce (1), and whether DC or CO is the preferred primary procedure for treating TBI remains controversial. A number of studies have compared the short-term or long-term outcomes of the two approaches. Chen et al. (6) and Rush et al. (10) suggested a higher in-hospital mortality rate in the DC group. However, their studied population was limited to acute subdural hematoma (ASDH) only. In other study by Kinoshita et al. (4), the researchers suggested a higher delayed hemorrhage rate and significantly unfavorable outcome at 6 months after surgery in elderly TBI patients who underwent CO as compared with DC. On the contrary, in the propensity-matched study by Jehan et al. (2), the authors revealed that there was no difference in the mortality rate, adverse discharge disposition, and GOS at discharge between the DC and CO groups. However, they did find higher complication rates and ventilator days in patients from the DC group. Meanwhile, the results from the DECRA (11) and RESCUEicp (12) trials suggested that DC can be performed as a life-saving procedure for TBI patients with significant brain swelling and refractory intracranial hypertension after CO as it provides the space to expand the edematous brain tissue and lower the ICP, but with higher severe disability and a persistent vegetative status rate. Although these aforementioned studies had investigated the prognosis of DC or CO comprehensively, they had mainly focused on patient survival and physical functions, neglecting the neuropsychological prognosis. Therefore, our study was trying to use multiple neuropsychological scales to measure the long-term outcomes of DC vs. CO as the primary procedure for TBI patients.

Besides the sensory and motor deficits caused by TBI, cognitive and behavioral changes are more likely associated with long-term disability. About 65% of moderate to severe TBI patients experienced long-term cognitive impairment (13), which may lead to limitations in vocational, recreational, and social areas of daily functions. A previous study had confirmed that white matter damage, such as diffuse axonal injury (DAI) caused by acceleration/deceleration forces from primary injury, is one of the important determinants of cognitive impairment of TBI (14). Secondary injuries, such as ischemia, changes in cerebral perfusion pressure (CPP), brain swelling, and inflammation, also relate to long-term outcomes (15). In addition, according to our research, patients in the DC group had significantly worse cognitive function compared with their counterparts in the CO group during the rehabilitation period, which may indicate that the different surgical procedures may have an impact on the neuropsychological outcomes too. In addition, that the resultant complications occur days to months after DC definitively plays an important role in the TBI patient's rehabilitation. First, unfavorable outcomes had been observed in a previous study following expansion of hemorrhagic contusions in TBI patients (4), and one theory is that relief of the tamponade effect with bone removal may contribute to it (16). Apart from this, post-traumatic hydrocephalus (PTH) is another common complication of DC because of the perturbation of cerebrospinal fluid (CSF) flow dynamics, and it has been associated with poorer outcomes following TBI. Mazzini et al. had shown that PTH can influence the functional and behavioral outcomes, which may associate with cortical atrophy and hypoperfusion in the regional lobes (17). In our study, there were nine and three patients who underwent DC-developed shunt-dependent hydrocephalus in cohorts before and after matching, respectively. However, none of the patients in the CO group required this procedure. Unfortunately, there was no significant statistical difference compared to the DC group in the cohort after matching. The syndrome of the trephined is also a frequent, delayed complication of DC. Patients often present with motor weakness, cognitive deficits, language deficits, altered levels of consciousness, headache, psychosomatic disturbances, seizures or electroencephalographic changes, and cranial nerve deficits, which typically arise weeks to months following DC. Changes in atmospheric pressure, decreases in cerebral blood flow, altered CSF circulation, and cerebral metabolism deficiency have been proposed to explain the pathophysiology underlying the syndrome (18). Nevertheless, cranioplasty can lead to significant, quantifiable functional improvement in the majority of patients (19). In the absence of contraindications, the Chinese Head Trauma Committee recommends undergoing cranioplasty early since it has a better prognosis than later cranioplasty. However, until 2016, surgical cranioplasty in China was usually performed at least 6 months after DC (20). On the other side, additional surgery can increase the incidence of complications and affect the patient's prognosis.

Daily functioning is also an aspect that responds to the long-term outcomes in patients with TBI. From the results of our study on the ADL, patients in both groups had varying degrees of functional decline. However, in the cohort after matching, the PSMS scores were close, with only the IADL and the ADL total showing that patients in the CO group had better daily functioning than those in the DC group. Furthermore, it has been proposed that cognitive function and self-awareness of individuals with TBI are good predictors of IADL functional performance (21). This also proves that patients who underwent CO have a similar physical outcome but better long-term outcomes in terms of cognition compared to patients in DC.

### Limitations

The present study has several limitations that should be mentioned. Our research only included patients who were able to complete the neuropsychological assessments alone or with guidance; therefore, the individuals unable to conduct the test because of aphasia, visual or hearing impairments, and very severe cognitive deficits or being in a vegetative state were out of this range. On the other side, our research had a small sample size and some patients in the CO group were not fully matched, which may have caused selection bias. In the meantime, our research was from a single center and lacks generalizability. In addition, we also could not trace whether there are obvious factors before and during surgery as well as factors such as personal preferences, which lead surgeons to choose a specific procedure.

In summary, future research studies are needed to verify our findings, preferentially by using more accurate neurocognitive battery tests in larger sample sizes, which may contribute to more intensive studies being conducted in this area.

## Conclusions

Our current study demonstrated that after balancing the patient demographics, cause of injury, TBI subtype, TBI severity, CT findings, surgery side, and interval from TBI, patients who underwent CO achieved better long-term outcomes in cognitive and daily functions compared to DC patients. Although DC is a radical surgery and has not shown superiority in the neuropsychological prognosis, it is still considered as a life-saving procedure for TBI patients. For these reasons, we suggest that when surgeons are faced with a TBI patient requiring surgical involvement, they should consider not only the preoperative characteristics and injury severity but also the resultant complications and long-term functional outcomes. In addition, besides physical rehabilitation, TBI patients may need long-term support, including cognitive rehabilitation as well as social, vocational, and family support. This study provides a new direction for future randomized control trials to better understand the impact of both procedures on long-term patient outcomes, resulting in better physical and neuropsychological prognosis for TBI patients.

## Data Availability Statement

The raw data supporting the conclusions of this article will be made available by the authors, without undue reservation.

## Author Contributions

ZG: conceptualization and writing–original draft. WD: methodology and psychological assessment. YC and DC: date curation and software. JC: writing–review and editing and supervision. All authors contributed to the article and approved the submitted version.

## Conflict of Interest

The authors declare that the research was conducted in the absence of any commercial or financial relationships that could be construed as a potential conflict of interest.

## Publisher's Note

All claims expressed in this article are solely those of the authors and do not necessarily represent those of their affiliated organizations, or those of the publisher, the editors and the reviewers. Any product that may be evaluated in this article, or claim that may be made by its manufacturer, is not guaranteed or endorsed by the publisher.
